# Associations of early changes in lung ultrasound aeration scores and mortality in invasively ventilated patients: a post hoc analysis

**DOI:** 10.1186/s12931-024-02893-0

**Published:** 2024-07-08

**Authors:** Jante S. Sinnige, Daan F. L. Filippini, Laura A. Hagens, Nanon F. L. Heijnen, Ronny M. Schnabel, Marcus J. Schultz, Dennis C. J. J. Bergmans, Lieuwe D. J. Bos, Marry R. Smit

**Affiliations:** 1 Department of Intensive Care, Amsterdam UMC, University of Amsterdam, Meibergdreef 9, Amsterdam, 1105 AZ the Netherlands; 2 Department of Intensive Care, Maastricht UMC+, Maastricht University, Maastricht, 6229 HX The Netherlands; 3 School of Nutrition and Translational Research in Metabolism (NUTRIM), Maastricht University, Maastricht, 6229 ER The Netherlands; 4 Mahidol Oxford Tropical Medicine Research Unit (MORU), Mahidol University, Bangkok, 10400 Thailand; 5 Nuffield Department of Medicine, University of Oxford, Oxford, OX3 7BN UK; 6 Department of Anesthesia, General Intensive Care and Pain Management, Division of Cardiothoracic and Vascular Anesthesia & Critical Care Medicine, Medical University of Vienna, Vienna, Austria; 7 Department of Pulmonology, Amsterdam UMC, University of Amsterdam, Amsterdam, 1105 AZ The Netherlands; 8 Laboratory of Experimental Intensive Care and Anaesthesiology (L.E.I.C.A.), University of Amsterdam, Amsterdam, 1105 AZ The Netherlands

**Keywords:** Lung ultrasound, Mortality, Intensive Care medicine, Longitudinal research

## Abstract

**Background:**

Lung ultrasound (LUS) in an emerging technique used in the intensive care unit (ICU). The derivative LUS aeration score has been shown to have associations with mortality in invasively ventilated patients. This study assessed the predictive value of baseline and early changes in LUS aeration scores in critically ill invasively ventilated patients with and without ARDS (Acute Respiratory Distress Syndrome) on 30- and 90-day mortality.

**Methods:**

This is a post hoc analysis of a multicenter prospective observational cohort study, which included patients admitted to the ICU with an expected duration of ventilation for at least 24 h. We restricted participation to patients who underwent a 12-region LUS exam at baseline and had the primary endpoint (30-day mortality) available. Logistic regression was used to analyze the primary and secondary endpoints. The analysis was performed for the complete patient cohort and for predefined subgroups (ARDS and no ARDS).

**Results:**

A total of 442 patients were included, of whom 245 had a second LUS exam. The baseline LUS aeration score was not associated with mortality (1.02 (95% CI: 0.99 – 1.06), *p* = 0.143). This finding was not different in patients with and in patients without ARDS. Early deterioration of the LUS score was associated with mortality (2.09 (95% CI: 1.01 – 4.3), *p* = 0.046) in patients without ARDS, but not in patients with ARDS or in the complete patient cohort.

**Conclusion:**

In this cohort of critically ill invasively ventilated patients, the baseline LUS aeration score was not associated with 30- and 90-day mortality. An early change in the LUS aeration score was associated with mortality, but only in patients without ARDS.

**Trial registration:**

ClinicalTrials.gov, ID NCT04482621.

**Supplementary Information:**

The online version contains supplementary material available at 10.1186/s12931-024-02893-0.

## Background

Acute respiratory distress syndrome (ARDS) is characterized by bilateral pulmonary opacities on imaging, accompanied by hypoxemia within one week of a known clinical insult [1]. The presence of ARDS in invasively ventilated patients is associated with high mortality and morbidity [2]. The pulmonary edema, present in ARDS, can be quantified at the bedside by using the chest X-ray based Radiographic Assessment of Lung Edema (RALE) score or by estimating extravascular lung water with a transpulmonary thermodilution method [3, 4]. These techniques showed to have predictive value for mortality in ARDS patients [5–8]. However, are invasive or require radiation.

Lung ultrasound (LUS) is a non-invasive, easy to learn, bedside technique that does not require radiation. It can accurately quantify the extent of pulmonary edema through the LUS aeration score [9–11]. The LUS aeration score was identified as a predictor for mortality by several studies in adult patients with COVID-19 [12–14]. However, the predictive value of the LUS aeration score remains unknown in ARDS patients without COVID-19 or in invasively ventilated patients without ARDS on mortality. Furthermore, the previous studies only assessed the predictive value of LUS aeration scores on admission, while early changes in the extent of pulmonary edema could be of additional predictive value [15].

In this study, we assessed the association of the baseline LUS aeration score and of early changes in LUS aeration scores with mortality in critically ill invasively ventilated patients with and without ARDS. We hypothesized that a both a higher baseline LUS aeration score and an early increase in LUS aeration score are associated with higher 30 and 90-day mortality in patients with and without ARDS.

## Methods

This is a post hoc analysis of patients included in the ‘Diagnosis of Acute Respiratory disTress Syndrome’ (DARTS) project. This multicentre prospective observational cohort study recruited patients from March 27, 2019 until February 27, 2021 in two hospitals in the Netherlands; (1) Amsterdam University Medical Center (Amsterdam UMC), location Academic Medical Center (AMC) and (2) Maastricht University Medical Center + (MUMC +). The protocol was approved by the institutional ethics committees of both centers (ref: W18_311 #18.358 and 2019–1137) and patients or legal representatives provided deferred consent for the use of data. The protocol of the DARTS project was previously published [16].

### Population

Adult patients were included in the study if they were admitted to a participating ICU and were expected to be invasively ventilated for at least 24 h. Patients were excluded if they had received invasive ventilation more than 48 h in the last 7 days or were receiving invasive ventilation by a tracheostomy. This post hoc analysis was restricted to patients who received a 12-region LUS exam at inclusion and had data on 30-day mortality available. ARDS was diagnosed by an expert panel according to the Berlin criteria using chest imaging, clinical parameters, and blood gas analysis [17].

### Lung ultrasound

**Fig. 1:**
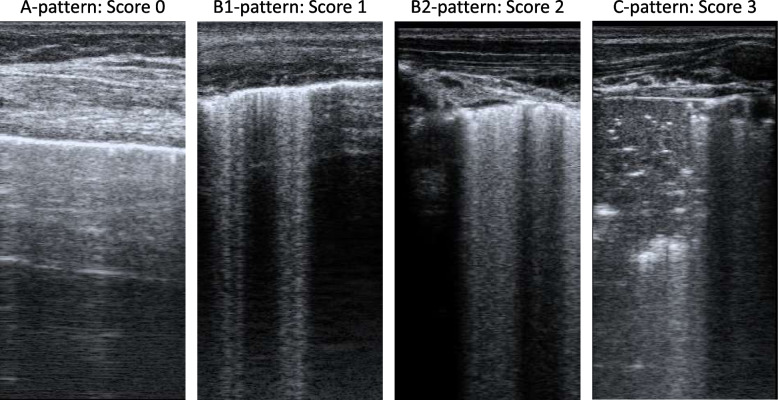
A-pattern; repeating horizontal A-lines parallel to the pleural line. B1-pattern; three or more vertical B-lines starting from the pleural line and reaching the bottom of the screen cover ≤ 50% of the pleural line (score 1). B2-pattern; B-lines cover ≥ 50% of the pleural line. C-pattern; consolidated lung [19]

Patients received a 12-region LUS exam at inclusion and 24 h after inclusion by three dedicated investigators [16, 18]. During the LUS exam, patients were positioned in supine position. LUS exams were performed with a linear probe using the clinically available ultrasound device. The use of other probes was allowed when the linear probe did not generate a sufficient image. Patients were scanned at two anterior, two lateral and two posterior locations per hemi thorax [16]. Each LUS image was scored as ‘0’ when A-lines were present, as ‘1’ when more than two B-lines covered < 50% of the pleura, as ‘2’ when B-lines covered > 50% of the pleura, and as ‘3’ when a consolidation of the lung was present (Fig. 1). If a lung region could not be scored or scanned (e.g., subcutaneous emphysema, chest drains, or wounds) the mean LUS aeration score of the same lung region (anterior, lateral, or posterior) was used as a substitute. Patients with more than four missing regions were excluded from this analysis. The LUS aeration score was calculated as the sum of LUS aeration scores in the 12 regions and could range from 0–36.

A sensitivity analysis was conducted on the LUS aeration score with only anterolateral regions, as the posterior regions might contain less signal as they commonly present loss of aeration, and the anterolateral regions are easy to reach (LUS darts). The LUS aeration score for the anterolateral fields can range from 0–24. Patients with more than three regions missing were excluded from this sensitivity analysis.

The early changes in LUS aeration score were calculated by subtracting the LUS aeration score at inclusion from the LUS aeration score 24 h after inclusion. A negative score correlates with an improvement of the LUS aeration score as a positive score correlates with a deterioration of the LUS aeration score.

### Study endpoints

The primary endpoint of the study was the association between LUS aeration score at baseline and the 30 and 90-day mortality. Additional endpoints were (1) association between early changes and deterioration of the LUS aeration score and 30-day mortality, (2) differences in LUS aeration scores between the predefined subgroups (ARDS and no ARDS), (3) the association between the baseline LUS aeration score and ARDS severity, and (4) the association between the baseline and early changes of the anterolateral LUS aeration score and 30-day mortality. Endpoints were adjusted for age, gender and the Acute Physiology and Chronic Health Evaluation II (APACHE II) score as they are prognostic variables for outcomes in the general ICU population [3, 20].

### Statistical analysis

The DARTS project sample size was based on an expected sensitivity of 80% for the exhaled breath analyses, with a minimal acceptable lower confidence limit of 65%, requiring at least 52 ARDS patients. Given a predicted ARDS incidence of 10.4%, a total sample size of at least 500 patients was needed to meet the primary endpoint. We did not calculate a sample size or perform a power analysis for this post hoc analysis.

Continuous data was reported as mean with standard deviation (SD) or median with interquartile range (IQR), depending on the distribution of the data. Categorical data was reported as number with percentage. The respective appropriate test was used, either normal distributed (t-test) or non-normal distributed (Kruskal Wallis or Mann–Whitney U test). The statistical distribution of data was controlled by the visual assessment of histograms and Q-Q plots. Logistic regression was used to analyze the primary and secondary endpoints. Independent variables were assessed for multicollinearity using the variance inflation factor. A locally estimated scatterplot smoothing (LOESS) regression was employed to visualize the association between LUS aeration scores and mortality, aiming to assess the feasibility of categorization without relying on arbitrary cut-off values. Data was tested two-sided, a type I error below 5% was considered statistically significant. The analyses were performed using RStudio (version 4.2.1, R Foundation for Statistical Computing, Vienna, Austria).

## Results

### Study population

**Fig. 2:**
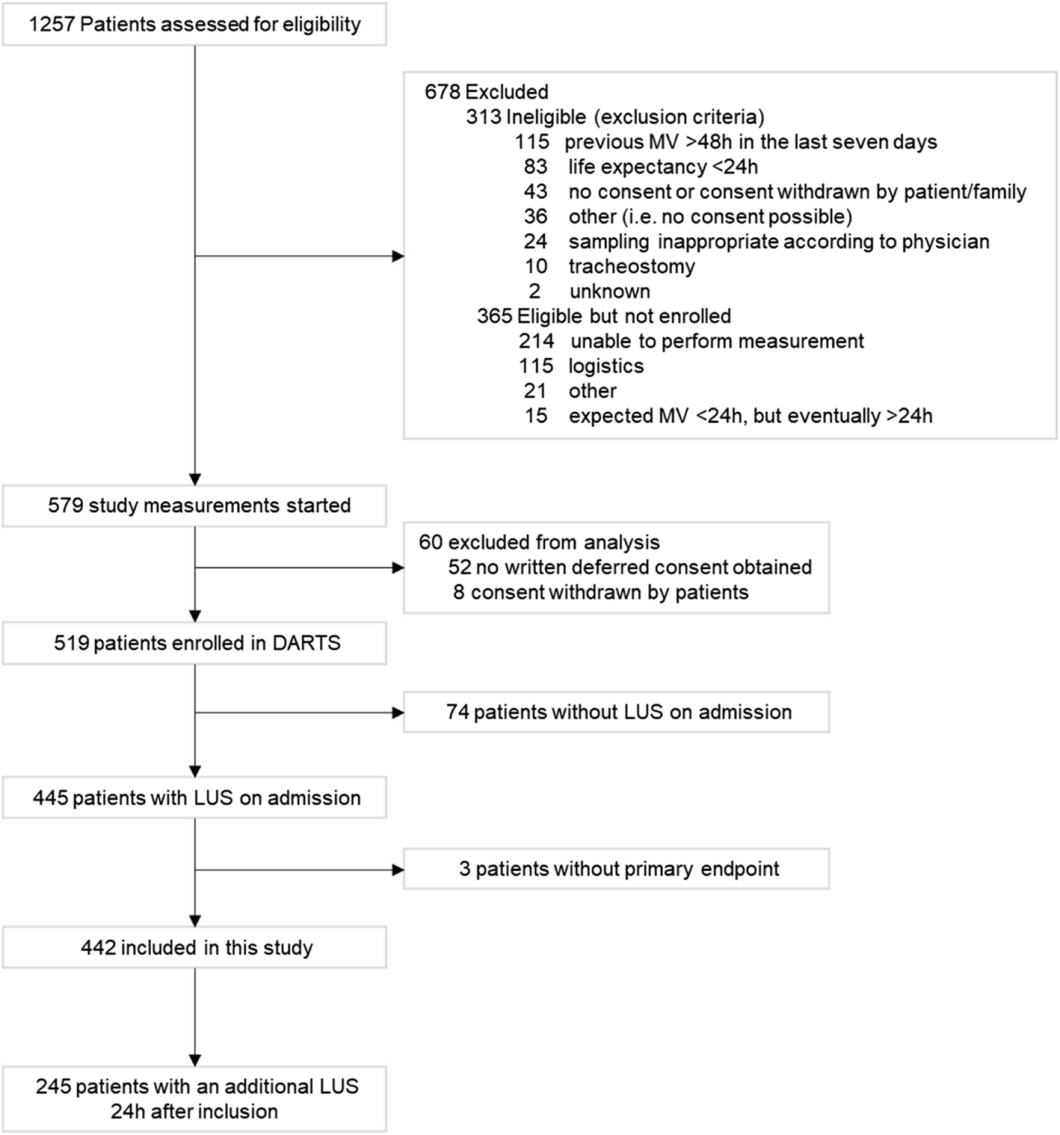
CONSORT figure of the patient enrolment in the DARTS consortium with additional exclusion criteria for the secondary analysis of this study. MV = Mechanically ventilated; DARTS = ‘Diagnosis of Acute Respiratory Distress Syndrome’ project [16]; LUS = Lung Ultrasound

A total of 442 (85%) of the 519 patients within the DARTS project had a LUS exam at inclusion and the primary endpoint available (Fig. 2, Table 1). ARDS was present in 152 (34%) of the patients and 171 (39%) patients were deceased by day 30. Patients who were deceased at day 30 were significantly older, had higher lactate levels, and had a higher APACHE II and Sequential Organ Failure Assessment (SOFA) score. Two hundred forty-five patients (55%) had a second LUS exam 24 h after inclusion and could be included in the analyses for the early changes in the LUS aeration score (Additional file 1).

**Table 1:** Baseline characteristics of patients at inclusion, stratified by survivors and deceased at day 30

	**All** ***n* = 442**	**Survived at 30 Days** ***n* = 271**	**Deceased at 30 Days** ***n* = 171**	***p*-Value**
**Demographics**				
Age (years (SD))	62 (15)	60 (15)	66 (13)	< 0.001*
Male (%)	293 (66)	180 (66)	113 (66)	1.000
BMI (kg m^−2^)	26.2 (23.5, 29.7)	26.2 (23.6, 29.7)	26.1 (23.4, 29.8)	0.783
APACHE II score	20 (15, 26)	20 (15, 24)	23 (16, 26)	< 0.001*
SOFA score	9 (7, 11)	9 (7, 11)	10 (7, 12)	0.014*
Lactate (mmol/L)	1.7 (1.2, 2.5)	1.6 (1.2, 2.2)	1.9 (1.3, 3.0)	0.002*
**Admission characteristics**				
ICU stay at inclusion (days)	1.0 (0.0, 1.0)	1.0 (0.0, 1.0)	1.0 (0.0, 1.0)	0.907
Admission type (%)				0.020*
Medical	327 (74)	200 (74)	127 (74)	
Emergency surgical	62 (14)	31 (11)	31 (18)	
Planned surgical	53 (12)	40 (15)	13 (8)	
COVID-19	47 (11)	29 (11)	18 (11)	1.000
**Respiratory**				
Hours of ventilation before inclusion (h)	21 (12, 28)	21 (12, 28)	20 (12, 28)	0.489
Maximum airway pressure (cmH_2_O)	20 (16, 25)	21 (16, 25)	20 (16, 25)	0.772
Driving pressure (cmH_2_O)	13 (9, 17)	13 (9, 18)	13 (9, 17)	0.682
PEEP (cmH_2_O)	8 (5, 10)	8 (5, 10)	8 (5, 10)	0.668
**ARDS**				
No ARDS	290 (66)	179 (66)	111 (65)	0.886
ARDS severity				0.586
Mild ARDS	21 (14)	12 (13)	9 (15)	
Moderate ARDS	81 (53)	47 (51)	34 (57)	
Severe ARDS	48 (32)	32 (35)	16 (27)	
Severity unavailble	2 (1)	1 (1)	1 (1)	
**Outcomes**				
ICU Length of stay (days)	7 (3, 13)	7 (3, 13)	6 (3, 14)	0.320
ICU mortality (%)	148 (34)	6 (2)	142 (85)	< 0.001*
**LUS aeration score**				
At baseline	7 (3, 13)	7 (2, 12)	8 (3, 14)	0.180
24 h after inclusion	8 (3, 15)	6 (2, 13)	10 (4, 16)	0.016*

^*^Significant difference. *SD* Standard Deviation, *BMI* Body Mass Index, *APACHE II*   Acute Physiology and Chronic Health Evaluation II, *SOFA* Sequential Organ Failure Assessment, *ICU* Intensive Care Unit, *COVID-19 *Coronavirus disease 2019, *h* Hours, *PEEP*  Positive End-Expiratory Pressure, *ARDS* Acute Respiratory Distress Syndrome, *LUS*  Lung ultrasound

### Baseline LUS aeration scores in patients with and without ARDS

**Fig. 3:**
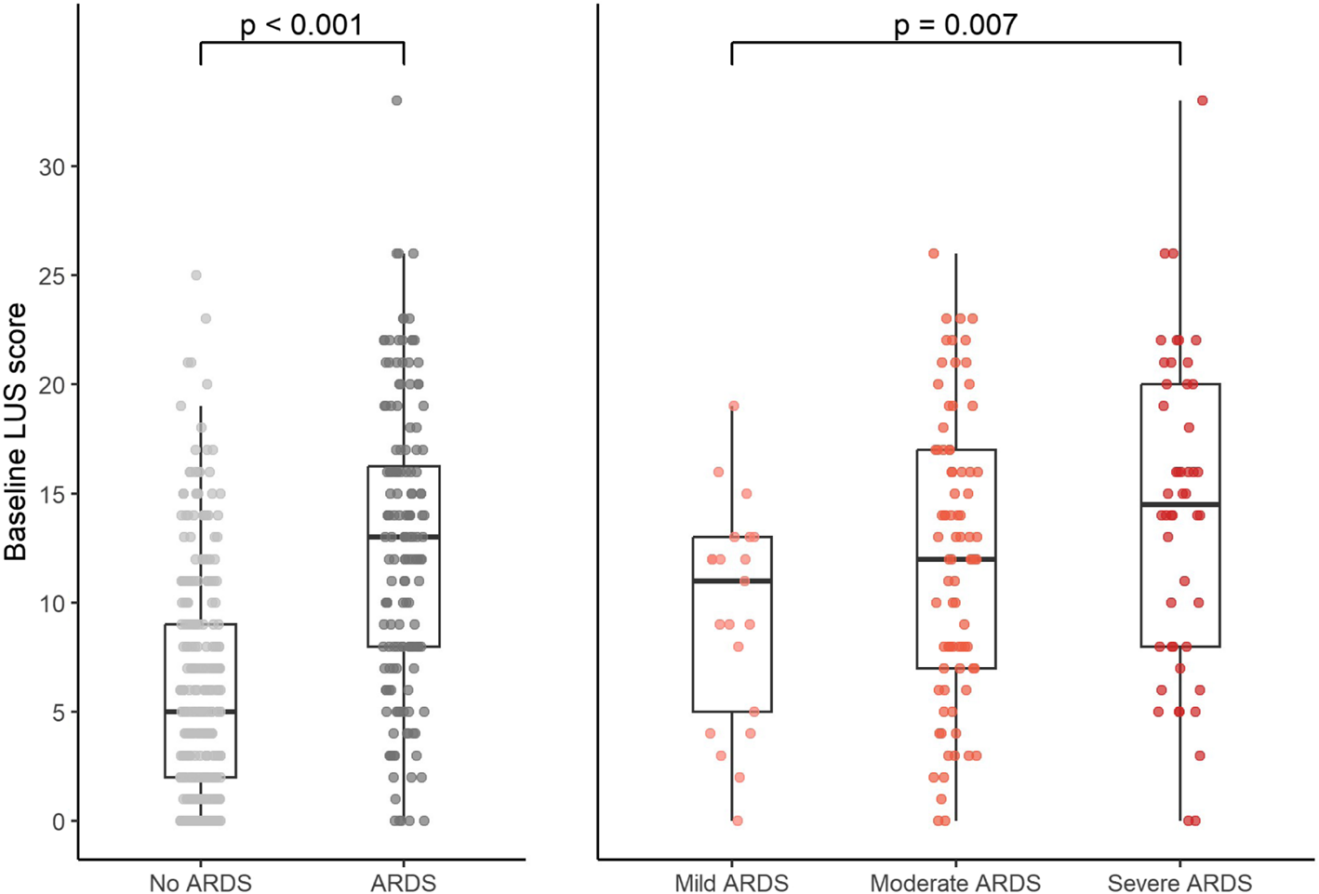
Differences in distributions of the baseline LUS aeration scores in the predefined groups. Individual patients are displayed as single-coloured dots. When a significant difference was found, the p-value was displayed above the figure. ARDS = Acute Respiratory Distress Syndrome; LUS = Lung Ultrasound

**Fig. 4:**
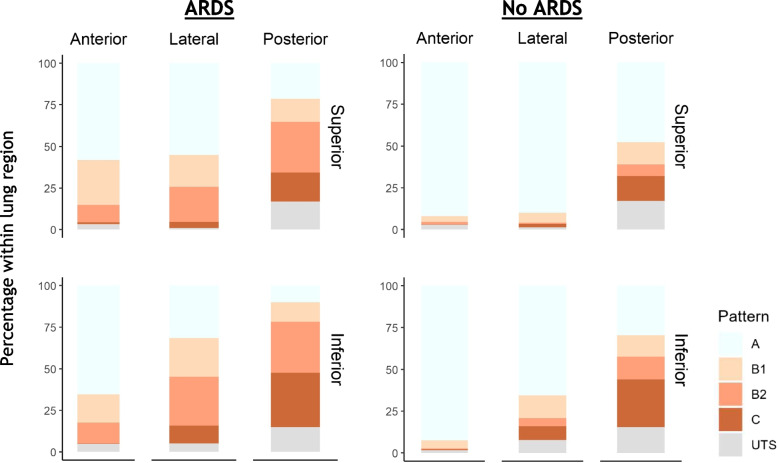
Distribution of the LUS patterns in patients with and without ARDS at baseline. The scores of the left and right lung are combined resulting in six regions per group. ARDS = acute respiratory distress syndrome; LUS = lung ultrasound, UTS = unable to score

The median baseline LUS aeration score was significantly higher in patients with ARDS in comparison to patients without ARDS (13 [IQR 8, 16] vs. 5 [IQR 2, 9], *p* < 0.001, Fig. 3, Additional file 2). Patients with severe ARDS had a significantly higher median baseline LUS aeration scores than patients with mild ARDS (15 [IQR 8, 20] vs. 11 [IQR 5, 13], *p* = 0.007). The distribution of LUS scoring in the six regions of the lungs are presented in Fig. 4, stratified for patients with and without ARDS.

### Association between baseline LUS aeration score and mortality

**Fig. 5:**
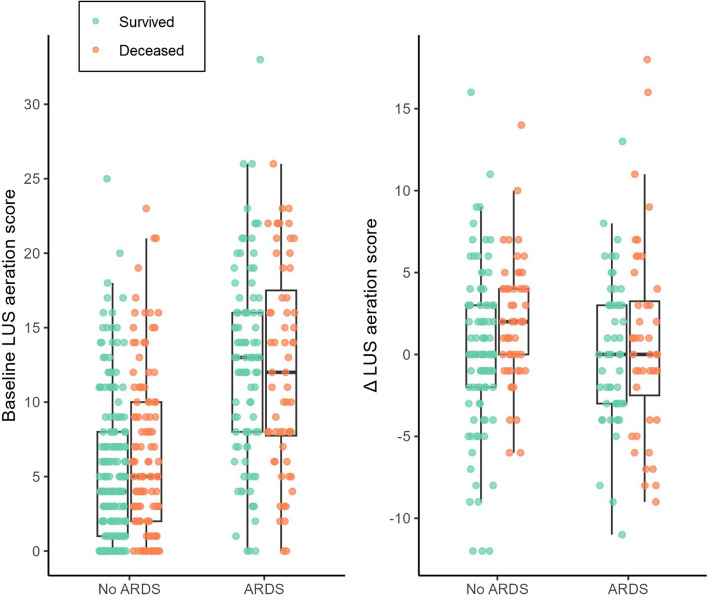
Differences in the baseline and early changes (Δ) of the LUS aeration scores in survivors and deceased patients with and without ARDS. ARDS = Acute Respiratory Distress Syndrome; LUS = Lung Ultrasound

The baseline LUS aeration scores in patients with and without ARDS were not associated with mortality at day 30 and day 90 in invasively ventilated patients on the ICU (Tables 2 and  3, Fig. 5). The results remained consistent across both univariable and multivariable analyses. Visualization of the individual data points did not result in a cut-off value to dichotomize the baseline LUS aeration score to improve these results (Additional file 3-5). The results remained consistent when only anterolateral regions were analysed (Additional file 6).

**Table 2:** Association between baseline LUS aeration scores and 30-day mortality in all patients and the predefined subgroups (No ARDS and ARDS), values are obtained using logistic regression and are presented as OR with 95% CI, indicating the increase per 1 point increment of the predictor variable

	**All** ***n* = 442**		**No ARDS** ***n* = 290**		**ARDS** ***n* = 152**	
	OR (CI)	*p*-Value	OR (CI)	*p*-Value	OR (CI)	*p*-Value
**Univariable analysis**						
LUS aeration score	1.02 (0.99 – 1.05)	0.193	1.04 (1 – 1.09)	0.059	1 (0.95 – 1.05)	0.942
**Multivariable analysis**						
LUS aeration score	1.02 (0.99 – 1.06)	0.143	1.03 (0.98 – 1.08)	0.227	1.02 (0.97 – 1.07)	0.505

Age, gender, and the APACHE II score were used in the multivariable analysis. *ARDS* Acute Respiratory Distress Syndrome, *OR* Odds Ratio, *CI* 95% Confidence Intervals, *LUS* Lung Ultrasound, *APACHE II* Acute Physiology and Chronic Health Evaluation II

**Table 3:** Association between baseline LUS aeration scores and 90-day mortality in all patients and the predefined subgroups (No ARDS and ARDS), values are obtained using logistic regression and are presented as OR with 95% CI, indicating the increase per 1 point increment of the predictor variable

	**All** ***n* = 441**		**No ARDS** ***n* = 289**		**ARDS** ***n* = 152**	
	OR (CI)	*p*-Value	OR (CI)	*p*-Value	OR (CI)	*p*-Value
**Univariable analysis**						
LUS aeration score	1.01 (0.99 – 1.04)	0.32	1.02 (0.98 – 1.07)	0.325	1.01 (0.96 – 1.06)	0.769
**Multivariable analysis**						
LUS aeration score	1.02 (0.99 – 1.05)	0.223	1.01 (0.96 – 1.06)	0.707	1.03 (0.97 – 1.08)	0.343

Age, gender, and the APACHE II score were used in the multivariable analysis. *ARDS* Acute Respiratory Distress Syndrome, *OR* Odds Ratio, *CI* 95% Confidence Intervals, *LUS* Lung Ultrasound, *APACHE II* Acute Physiology and Chronic Health Evaluation II

### Association between early LUS changes and survival

In patients without ARDS (*n* = 75), deterioration of LUS aeration score was associated with mortality (Table 4). This relation remained in the multivariable analysis. However, there was no association between mortality and the deterioration of LUS aeration score in patients with ARDS, or in all patients in the multivariable analysis. Furthermore, the early changes in the LUS aeration scores and analysis of anterolateral fields did not have any additional predictive value in across patients and in the predefined subgroups (Fig. 5, Additional file 7).

**Table 4:** Association between early changes (Δ) in the LUS aeration scores and 30-day mortality in all patients and the predefined subgroups (No ARDS and ARDS), values are obtained using logistic regression and are presented as OR with 95% CI, indicating the increase per 1 point increment of the predictor variable in the Δ LUS analysis. Δ LUS > 0 indicates a deterioration of the LUS aeration score

	**All** ***n* = 245**		**No ARDS** ***n* = 150**		**ARDS** ***n* = 95**	
	OR (CI)	*p*-Value	OR (CI)	*p*-Value	OR (CI)	*p*-Value
**Univariable analysis**						
Δ LUS	1.06 (1 – 1.12)	0.037	1.1 (1.01 – 1.19)	0.022	1.03 (0.95 – 1.11)	0.491
Δ LUS > 0	1.83 (1.09 – 3.07)	0.022	2.58 (1.29 – 5.14)	0.007	1.2 (0.54 – 2.71)	0.654
**Multivariable analysis**						
Δ LUS	1.05 (0.99 – 1.12)	0.077	1.09 (1 – 1.19)	0.052	1.04 (0.95 – 1.13)	0.402
Δ LUS > 0	1.58 (0.92 – 2.71)	0.099	2.09 (1.01 – 4.3)	0.046	1.23 (0.52 – 2.91)	0.644

Age, gender, and the APACHE II score were used in the multivariable analysis. *ARDS* Acute Respiratory Distress Syndrome, *OR* Odds Ratio, *CI* 95% Confidence Intervals, *LUS* Lung Ultrasound, *APACHE II* Acute Physiology and Chronic Health Evaluation II

## Discussion

In this post hoc analysis of the DARTS project, we did not find an association between the baseline LUS aeration scores and 30- and 90-day mortality in invasively ventilated ICU patients and in the predefined ARDS subgroups. For early changes of the LUS aeration score, we did find that deterioration of the LUS aeration score in patients without ARDS was associated with 30-day mortality. However, this association was not found in ARDS patients nor in the whole cohort.

In the context of patients with ARDS, several studies assessed the predictive value of the LUS aeration score on mortality, but predominately in COVID-19 patients. While some of these studies showed an association between mortality and the LUS aeration score at baseline [12, 14, 21], other studies did not find this association [13, 22]. In addition to these contradictory findings, there is considerable variation in the timing of the LUS exam across these studies. Some studies conduct the exam upon admission, while another study performed the LUS exam seven days after admission. The studies using a larger timeframe from admission seem to find a better association between the LUS aeration score and mortality, potentially explaining why we did not find predictive value of the baseline LUS aeration score and early changes in the LUS aeration score in ARDS patients on mortality.

It is noteworthy that within the DARTS project, a similar study assessed the predictive value of the radiography-based RALE score on mortality in patients with and without ARDS [5]. This study showed that the early changes in the RALE score have predictive value for 30-day mortality in patients with ARDS, but not in patients without ARDS. Discrepancies in the findings between this and our study may arise from the differences in assessment of lung edema between the two imaging modalities. LUS has a tomographic approach, is sensitive to changes in lung aeration and typically scans a subpleural layer of the lung. On the other hand, chest X-ray (CXR) acquires a two-dimensional image of the entire lung, is less sensitive for changes in aeration than LUS and therefore probably requires more edema for the RALE score to increase [23]. Furthermore, our study cohort is a different patient group because the LUS exams were performed per protocol in the DARTS project, while the CXRs were performed on clinical indication. Studies on the RALE score as a predictive tool on mortality in ventilated ICU patients with ARDS show conflicting results, similar to the LUS aeration score [3, 5, 6, 24–27].

A strength of this prospective study is the large sample size containing multiple LUS exams per patient. Furthermore, unlike previous studies that mainly concentrated on the predictive value of LUS aeration score on mortality in COVID-19 patients, only 11% of the patients in this study were tested positive for SARS-CoV-2. This makes the findings of this study more generalizable for the ICU population. Additionally, LUS knows a high inter observer agreement [28]. Finally, in the current study, ARDS diagnosis was performed by a panel of experts, mitigating the typical challenges associated with substantial inter-observer variability in diagnosing ARDS [17]. A potential limitation of this study is the relatively short follow-up period of 24 h between the first and second LUS exam. This could have attributed to the absence of differences in the early changes of the LUS aeration score among ARDS patients, as severe pulmonary distress might not resolve or decrease within 24 h. Lastly, the study did not incorporate ventilator-free days as an endpoint, and therefore, the predictive value of LUS for duration of ventilation remains unknown.

This is the first study to highlight the predictive potential of LUS in determining mortality at day 30 in invasively ventilated patients without ARDS. While baseline LUS aeration scores did not demonstrate an association with mortality, such association was found in the early changes analysis with a repeated LUS exam after 24 h. After further validation of these findings, early changes in LUS aeration scores might serve as a potential indicator for predictive enrichment or as an early sign of treatment response in invasively ventilated patients without ARDS. Moving forward, the present findings should be externally validated and additional research on the timing of the LUS exam in invasively ventilated patients is warranted. Furthermore, incorporating subpleural consolidations and pleural abnormalities with the LUS aeration score could potentially improve the predictive value on mortality in ARDS patients.

## Conclusions

In conclusion, this study showed that early changes in the LUS aeration score have a predictive value for 30-day mortality in invasively ventilated ICU patients without ARDS. There was no association found between the baseline LUS aeration score and 30- and 90-mortality in patients with and without ARDS.

### Supplementary Information

Supplementary Material 1.

Supplementary Material 2.

Supplementary Material 3.

Supplementary Material 4.

Supplementary Material 5.

Supplementary Material 6.

Supplementary Material 7.

## Data Availability

The datasets used and/or analysed during the current study are available from the corresponding author on reasonable request.

## Abbreviations

LUS: Lung ultrasound
ICU: Intensive Care Unit
ARDS: Acute Respiratory Distress Syndrome
RALE: Radiographic Assessment of Lung Edema
COVID-19: Coronavirus disease 2019
DARTS: Diagnosis of Acute Respiratory disTress Syndrome
Amsterdam UMC: Amsterdam University Medical Center
AMC: Academic Medical Center
MUMC: Maastricht University Medical Center
APACHE II: Acute Physiology and Chronic Health Evaluation II
SD: Standard Deviation
IQR: Interquartile range
LOESS: Locally estimated scatterplot smoothing
BMI: Body Mass Index
SOFA: Sequential Organ Failure Assessment
h: Hours
PEEP: Positive End-Expiratory Pressure
