# Supplementary material for: Associations of early changes in lung ultrasound aeration scores and mortality in invasively ventilated patients: a post hoc analysis
Source: Respir Res. 2024 Jul 8;25:268. doi: 10.1186/s12931-024-02893-0 (PMC11232207; doi:10.1186/s12931-024-02893-0)
Supplement: Supplementary file 1 — Supplementary Material 1. [file 12931_2024_2893_MOESM1_ESM.docx]

|  | **All**  **n = 442** | **One LUS exam**  **n = 197** | **Two LUS exam**  **n = 245** | **p-Value** |
| --- | --- | --- | --- | --- |
| **Demographics** |  |  |  |  |
| Age (years (SD)) | 62 (15) | 62 (14) | 62 (15) | 0.680 |
| Male (%) | 293 (66) | 135 (69) | 158 (65) | 0.429 |
| BMI (kg m^-2^) | 26.2 (23.5, 29.7) | 26.1 (23.3, 29.4) | 26.3 (23.7, 30.0) | 0.821 |
| APACHE II score | 20 (15, 26) | 20 (15, 26) | 20 (15, 25) | 0.976 |
| SOFA score | 9 (7, 11) | 9 (7, 11) | 9 (7, 11) | 0.442 |
| Lactate (mmol/L) | 1.7 (1.2, 2.5) | 1.6 (1.1, 2.2) | 1.8 (1.3, 2.6) | 0.046* |
| **Admission characteristics** |  |  |  |  |
| ICU stay at inclusion (days) | 1.0 (0.0, 1.0) | 1.0 (0.0, 1.0) | 1.0 (0.0, 2.0) | 0.005* |
| Admission type (%) |  |  |  | 0.047* |
| - Medical | 327 (74) | 139 (71) | 188 (77) |  |
| - Emergency surgical | 62 (14) | 26 (13) | 36 (15) |  |
| - Planned surgical | 53 (12) | 32 (16) | 21 (9) |  |
| COVID-19 | 47 (11) | 18 (9) | 29 (12) | 0.447 |
| **Respiratory** |  |  |  |  |
| Hours of ventilation before inclusion (h) | 21 (12, 28) | 21 (11, 26) | 21 (13, 30) | 0.176 |
| Maximum airway pressure (cmH_2_O) | 20 (16, 25) | 20 (16, 25) | 21 (17, 25) | 0.114 |
| Driving pressure (cmH_2_O) | 13 (9, 17) | 13 (8, 16) | 14 (10, 18) | 0.126 |
| PEEP (cmH_2_O) | 8 (5, 10) | 8 (5, 10) | 8 (5, 10) | 0.504 |
| **ARDS** |  |  |  |  |
| No ARDS | 290 (66) | 140 (71) | 150 (61) | 0.039* |
| ARDS severity |  |  |  | 0.996 |
| - Mild ARDS | 21 (14) | 8 (14) | 13 (14) |  |
| - Moderate ARDS | 81 (53) | 30 (54) | 51 (54) |  |
| - Severe ARDS | 48 (32) | 18 (32) | 30 (32) |  |
| - Severity unavailble | 2 (1) | 1 (1) | 1 (1) |  |
| **Outcomes** |  |  |  |  |
| ICU Length of stay (days) | 7 (3, 13) | 4 (2, 10) | 9 (5, 15) | <0.001* |
| ICU mortality (%) | 148 (34) | 64 (33) | 84 (35) | 0.777 |
| **LUS aeration score** |  |  |  |  |
| At baseline | 7 (3, 13) | 6 (3, 12) | 7 (3, 14) | 0.135 |
| 24 hours after inclusion | 8 (3, 15) | NA | 8 (3, 15) | NA |
| **Additional file 1**. Baseline characteristics of patients at inclusion, stratified for patients with only a LUS exam at baseline and patients with a LUS exam at baseline and after 24 hours. *significant difference. SD = Standard Deviation; BMI = Body Mass Index; APACHE II = Acute Physiology and Chronic Health Evaluation II; SOFA = Sequential Organ Failure Assessment; ICU = Intensive Care Unit; COVID-19 = coronavirus disease 2019; h = hours; PEEP = Positive End-Expiratory Pressure; ARDS = Acute Respiratory Distress Syndrome. | | | | |

Additional file 1 - Baseline characteristics, stratified for number of LUS exams
