# Supplementary material for: Associations of early changes in lung ultrasound aeration scores and mortality in invasively ventilated patients: a post hoc analysis
Source: Respir Res. 2024 Jul 8;25:268. doi: 10.1186/s12931-024-02893-0 (PMC11232207; doi:10.1186/s12931-024-02893-0)
Supplement: Supplementary file 2 — Supplementary Material 2. [file 12931_2024_2893_MOESM2_ESM.docx]

|  | **No ARDS**  **n = 290** | **ARDS**  **n = 152** | **p-Value** |
| --- | --- | --- | --- |
| **Demographics** |  |  |  |
| Age (years (SD)) | 62 (15) | 62 (14) | 0.821 |
| Male (%) | 192 (66) | 101 (66) | 1.000 |
| BMI (kg m^-2^) | 26.1 (23.0, 29.8) | 26.3 (23.8, 29.8) | 0.437 |
| APACHE II score | 20 (15, 26) | 20 (15, 24) | 0.017* |
| SOFA score | 9 (7, 11) | 9 (7, 11) | 0.083 |
| Lactate (mmol/L) | 1.6 (1.1, 2.5) | 1.8 (1.3, 2.5) | 0.109 |
| **Admission characteristics** |  |  |  |
| ICU stay at inclusion (days) | 1 (0, 1) | 1 (0, 2) | <0.001* |
| Admission type (%) |  |  | 0.001* |
| - Medical | 198 (68.3) | 129 (84.9) |  |
| - Emergency surgical | 50 (17.2) | 12 (7.9) |  |
| - Planned surgical | 42 (14.5) | 11 (7.2) |  |
| COVID-19 | 1 (0.3) | 46 (30.3) | <0.001* |
| **Respiratory** |  |  |  |
| Hours of ventilation before inclusion (h) | 21 (13, 30) | 21 (11, 28) | 0.717 |
| Maximum airway pressure (cmH_2_O) | 19 (15, 24) | 24 (17, 29) | <0.001* |
| Driving pressure (cmH_2_O) | 13 (9, 16) | 14 (9, 18) | 0.023* |
| PEEP (cmH_2_O) | 6.5 (5, 8) | 10.0 (8, 12) | <0.001* |
| **Outcomes** |  |  |  |
| ICU Length of stay (days) | 6 (3, 11) | 9 (4, 16) | <0.001* |
| ICU mortality (%) | 92 (32) | 56 (37) | 0.380 |
| **LUS aeration score** |  |  |  |
| At baseline | 5 (2, 9) | 13 (8, 16) | <0.001* |
| 24 hours after inclusion | 5 (2, 11) | 13 (8, 18) | <0.001* |
| **Additional file 2.** Baseline characteristics stratified for patients with and without ARDS. *significant difference. SD = Standard Deviation; BMI = Body Mass Index; APACHE II = Acute Physiology and Chronic Health Evaluation II; SOFA = Sequential Organ Failure Assessment; ICU = Intensive Care Unit; COVID-19 = coronavirus disease 2019; h = hours; PEEP = Positive End-Expiratory Pressure; ARDS = Acute Respiratory Distress Syndrome. | | | |

Additional file 2 - Baseline characteristics, stratified for ARDS and No ARDS
