# Supplementary material for: Associations of early changes in lung ultrasound aeration scores and mortality in invasively ventilated patients: a post hoc analysis
Source: Respir Res. 2024 Jul 8;25:268. doi: 10.1186/s12931-024-02893-0 (PMC11232207; doi:10.1186/s12931-024-02893-0)
Supplement: Supplementary file 4 — Supplementary Material 4. [file 12931_2024_2893_MOESM4_ESM.docx]

Additional file 4 - Baseline LUS aeration score and mortality in ARDS patients


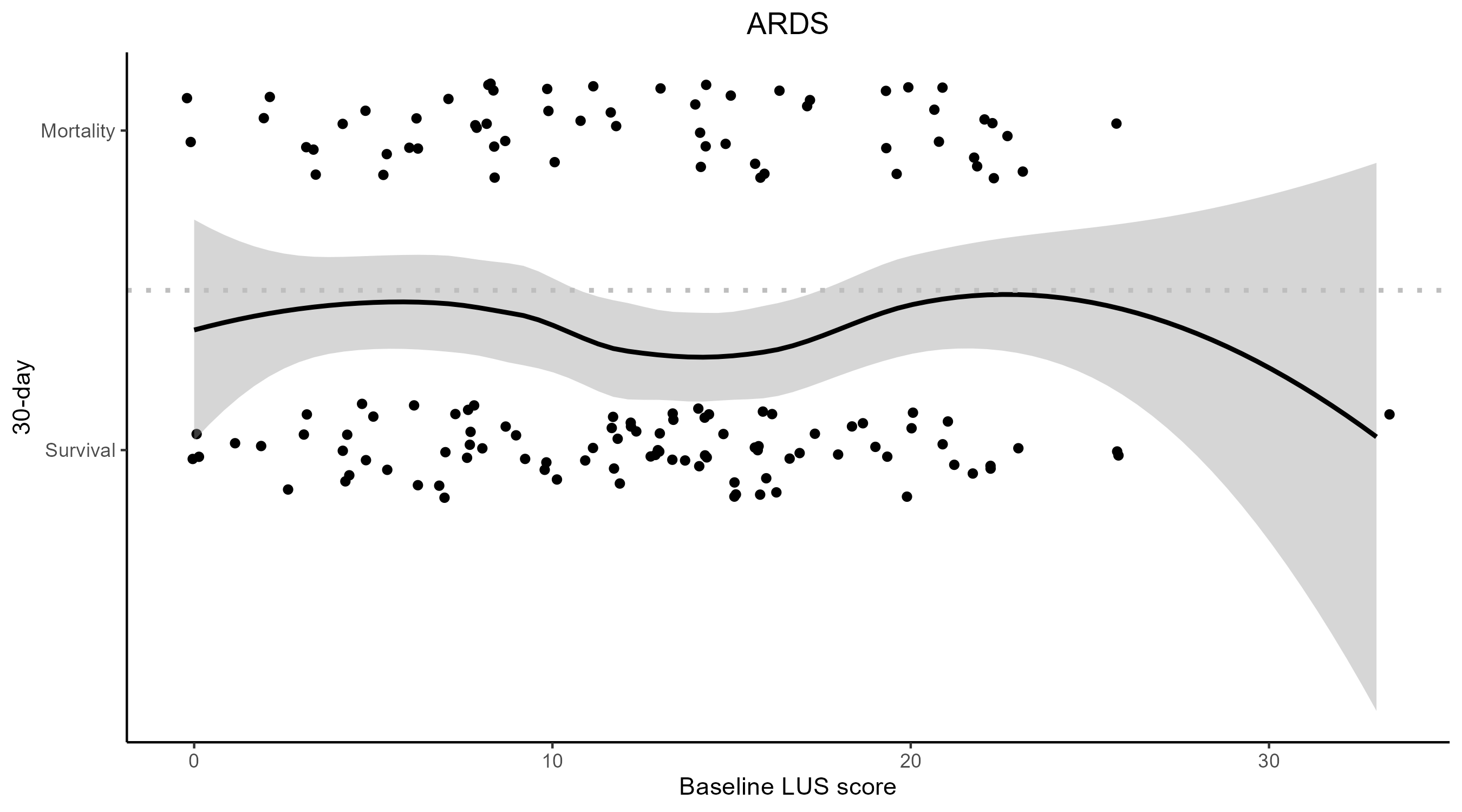


| **Additional file 4.** Individual patient data point of the baseline LUS aeration score presented as dots divided by 30 day mortality on the y-axis in ARDS patients. The black trend line is generated through LOESS regression, and the 95% confidence interval is represented in grey. LUS = Lung Ultrasound; ARDS = Acute Respiratory Distress Syndrome; LOESS = locally estimated scatterplot smoothing. |
| --- |
