# Supplementary material for: Associations of early changes in lung ultrasound aeration scores and mortality in invasively ventilated patients: a post hoc analysis
Source: Respir Res. 2024 Jul 8;25:268. doi: 10.1186/s12931-024-02893-0 (PMC11232207; doi:10.1186/s12931-024-02893-0)
Supplement: Supplementary file 6 — Supplementary Material 6. [file 12931_2024_2893_MOESM6_ESM.docx]

Additional file 6 - Anterolateral baseline LUS aeration score and 30 day mortality.

|  | **All**  **n = 437** | | **No ARDS**  **n = 289** | | **ARDS**  **n = 148** | |
| --- | --- | --- | --- | --- | --- | --- |
|  | OR (CI) | p-Value | OR (CI) | p-Value | OR (CI) | p-Value |
| **Univariable analysis** |  |  |  |  |  |  |
| Baseline LUS | 1.04 (0.99 – 1.1) | 0.088 | 1.1 (0.99 – 1.21) | 0.068 | 1.03 (0.96 – 1.11) | 0.385 |
| **Multivariable analysis** |  |  |  |  |  |  |
| Baseline LUS | 1.06 (1 – 1.11) | 0.041 | 1.07 (0.96 – 1.18) | 0.239 | 1.07 (0.99 – 1.15) | 0.092 |
| **Additional file 6**. Anterolateral baseline LUS aeration score and 30 day mortality. Age, gender and the APACHE II score were used in the multivariable analysis. ARDS = Acute Respiratory Distress Syndrome; OR = Odds Ratio; LUS = Lung Ultrasound; APACHE II = Acute Physiology and Chronic Health Evaluation II. | | | | | | |
