# Supplementary material for: Associations of early changes in lung ultrasound aeration scores and mortality in invasively ventilated patients: a post hoc analysis
Source: Respir Res. 2024 Jul 8;25:268. doi: 10.1186/s12931-024-02893-0 (PMC11232207; doi:10.1186/s12931-024-02893-0)
Supplement: Supplementary file 7 — Supplementary Material 7. [file 12931_2024_2893_MOESM7_ESM.docx]

Additional file 7 - Anterolateral early changes (Δ) in LUS aeration score and 30 day mortality

|  | **All**  **n = 245** | | **No ARDS**  **n = 150** | | **ARDS**  **n = 95** | |
| --- | --- | --- | --- | --- | --- | --- |
|  | OR (CI) | p-Value | OR (CI) | p-Value | OR (CI) | p-Value |
| **Univariable analysis** |  |  |  |  |  |  |
| Δ LUS | 1.06 (0.97 – 1.16) | 0.185 | 1.09 (0.96 – 1.24) | 0.203 | 1.05 (0.93 – 1.18) | 0.439 |
| Δ LUS > 0 | 1.17 (0.97 – 1.41) | 0.108 | 1.08 (0.85 – 1.36) | 0.534 | 1.32 (0.94 – 1.86) | 0.107 |
| **Multivariable analysis** |  |  |  |  |  |  |
| Δ LUS | 1.04 (0.95 – 1.15) | 0.367 | 1.08 (0.94 – 1.23) | 0.3 | 1.02 (0.89 – 1.17) | 0.77 |
| Δ LUS > 0 | 1.2 (0.99 – 1.46) | 0.067 | 1.1 (0.85 – 1.42) | 0.457 | 1.17 (0.8 – 1.69) | 0.418 |
| **Additional file 7**. Anterolateral early changes (Δ) in LUS aeration score and 30 day mortality. Age, gender and the APACHE II score were used in the multivariable analysis. ARDS = Acute Respiratory Distress Syndrome; OR = Odds Ratio; LUS = Lung Ultrasound; APACHE II = Acute Physiology and Chronic Health Evaluation II. | | | | | | |
